# Supplementary material for: The association between different body mass index levels and midterm surgical revascularization outcomes
Source: PLoS One. 2022 Sep 29;17(9):e0274129. doi: 10.1371/journal.pone.0274129 (PMC9522296; doi:10.1371/journal.pone.0274129)
Supplement: S1 Table — BMI, body mass index; MACCE, major adverse cardio-cerebrovascular events; ACS, acute coronary syndrome; CVA, cerebrovascular events. Data are presented as number and frequency. (DOCX) [file pone.0274129.s001.docx]

Supplementary Table 1. Event rates in the study cohort and by BMI category

|  | All patients  N=17751 | 18.5≤BMI<25  n= 5547 | 25≤BMI<30  n= 8091 | 30≤BMI<35  n= 3304 | 35≤BMI<40  n=661 | BMI≥40  n=148 |
| --- | --- | --- | --- | --- | --- | --- |
| All-cause mortality | 1838  (10.4) | 653  (11.8) | 783  (9.7) | 313  (9.5) | 66  (10.0) | 23  (15.5) |
| MACCE ( first-event) | 3540  (19.9) | 1163  (21.1) | 1547  (19.3) | 667  (20.2) | 132  (20.3) | 31  (21.1) |
| MACCE components  (first-event) | | | | | | |
| ACS | 1471  (8.3) | 433  (7.9) | 658  (8.1) | 304  (9.2) | 62  (9.6) | 10  (6.8) |
| CVA | 412  (2.3) | 140  (2.5) | 181  (2.3) | 80  (2.4) | 11  (1.7) | 0 |
| Death | 1661  (9.4) | 590  (10.7) | 708  (8.8) | 283  (8.6) | 59  (9) | 21  (14.3) |

BMI, body mass index; MACCE, major adverse cardio-cerebrovascular events; ACS, acute coronary syndrome; CVA, cerebrovascular events

Data are presented as number and frequency.
