# Supplementary material for: The association between different body mass index levels and midterm surgical revascularization outcomes
Source: PLoS One. 2022 Sep 29;17(9):e0274129. doi: 10.1371/journal.pone.0274129 (PMC9522296; doi:10.1371/journal.pone.0274129)
Supplement: S2 Table — HR, Hazard ratio; CI, Confidence interval; BMI, Body mass index; CKD, Chronic kidney disease; EF, Ejection fraction; SVD, Single vessel disease; VD, Vessel disease; ICU, Intensive care unit; MI, Myocardial infarction; COPD, Chronic obstructive pulmonary disease; CVA, Cerebrovascular accidents; TIA, Transient ischemic attack; PCI, Percutaneous coronary intervention; ACIE, Angiotensin converting enzyme inhibitor; ARB, Angiotensin II receptor blocker; ASA, Aspirin. (DOCX) [file pone.0274129.s002.docx]

Supplementary Table 2. The RCS model on All-cause mortality

| Variable | HR | 95%CI | P value |
| --- | --- | --- | --- |
| BMI | 1.03 | 1.02-1.05 | <0.001 |
| Age | 1.05 | 1.05-1.06 | <0.001 |
| Male | 2.51 | 2.16-2.93 | <0.001 |
| Diabetes | 1.45 | 1.31-1.61 | <0.001 |
| Hypertension | 1.35 | 1.21-1.51 | <0.001 |
| Hyperlipidemia | 0.91 | 0.81-1.02 | 0.108 |
| Positive family history | 0.99 | 0.88-1.1 | 0.791 |
| Current smoking | 1.21 | 1.05-1.4 | 0.011 |
| Opium | 1.21 | 1.04-1.4 | 0.013 |
| CKD | 1.74 | 1.54-1.96 | <0.001 |
| EF | 0.97 | 0.96-0.97 | <0.001 |
| Left main | 0.98 | 0.83-1.16 | 0.794 |
| VD | 1.26 | 1.12-1.41 | <0.001 |
| Graft number | 0.89 | 0.84-0.95 | 0.001 |
| ICU Hours | 1.001 | 1.001-1.001 | <0.001 |
| Off Pump | 1.07 | 0.87-1.33 | 0.5 |
| Recent MI | 0.99 | 0.83-1.18 | 0.903 |
| COPD | 1.3 | 1.04-1.63 | 0.024 |
| CVA/TIA | 1.53 | 1.3-1.8 | <0.001 |
| Previous PCI | 0.96 | 0.74-1.25 | 0.761 |
| ACEI/ARB | 0.96 | 0.87-1.07 | 0.484 |
| ASA/anti-platelets | 0.39 | 0.32-0.46 | <0.001 |
| Statins | 0.47 | 0.4-0.55 | <0.001 |
| Beta blockers | 0.56 | 0.48-0.65 | <0.001 |
| RCS1 | 1.98 | 1.92-2.04 | <0.001 |
| RCS2 | 0.87 | 0.85-0.89 | <0.001 |
| RCS3 | 0.86 | 0.84-0.87 | <0.001 |
| RCS4 | 0.98 | 0.96-1 | 0.013 |
| RCS5 | 0.99 | 0.97-1 | 0.029 |
| Constant | 0.01 | 0-0.02 | <0.001 |

HR, Hazard ratio; CI,Confidence interval; BMI, Body mass index; CKD, Chronic kidney disease; EF, Ejection fraction; SVD,Single vessel disease; VD, Vessel disease; ICU, Intensive care unit; MI, Myocardial infarction; COPD, Chronic obstructive pulmonary disease; CVA, Cerebrovascular accidents; TIA, Tranisent ischemic attack; PCI, Percutaneous coronary intervention; ACIE, Angiotensin converting enzyme inhibitor,; ARB, Angiotensin II receptor blocker; ASA, Aspirin
