# Supplementary material for: The association between different body mass index levels and midterm surgical revascularization outcomes
Source: PLoS One. 2022 Sep 29;17(9):e0274129. doi: 10.1371/journal.pone.0274129 (PMC9522296; doi:10.1371/journal.pone.0274129)
Supplement: S3 Table — MACCE, Major cardio-cerebrovascular events; HR, Hazard ratio; CI,Confidence interval; BMI, Body mass index; CKD, Chronic kidney disease; EF, Ejection fraction; SVD,Single vessel disease; VD, Vessel disease; ICU, Intensive care unit; MI, Myocardial infarction; COPD, Chronic obstructive pulmonary disease; CVA, Cerebrovascular accidents; TIA, Tranisent ischemic attack; PCI, Percutaneous coronary intervention; ACIE, Angiotensin converting enzyme inhibitor,; ARB, Angiotensin II receptor blocker; ASA, Aspirin. (DOCX) [file pone.0274129.s003.docx]

Supplementary Table 3. The RCS model on MACCE

| Variable | HR | 95% CI | P value |
| --- | --- | --- | --- |
| BMI | 1.02 | 1.01-1.03 | <0.001 |
| Age | 1.02 | 1.01-1.02 | <0.001 |
| Male | 1.27 | 1.16-1.39 | <0.001 |
| Diabetes | 1.29 | 1.2-1.39 | <0.001 |
| Hypertension | 1.28 | 1.19-1.39 | <0.001 |
| Hyperlipidemia | 0.92 | 0.85-0.99 | 0.033 |
| Positive family history | 1.02 | 0.94-1.1 | 0.67 |
| Current smoking | 1.1 | 0.99-1.22 | 0.08 |
| Opium | 1.11 | 0.99-1.23 | 0.066 |
| CKD | 1.45 | 1.32-1.59 | <0.001 |
| EF | 0.98 | 0.98-0.99 | <0.001 |
| Left main | 0.96 | 0.85-1.09 | 0.563 |
| VD | 1.09 | 1.01-1.18 | 0.029 |
| Graft number | 0.92 | 0.88-0.97 | 0.001 |
| ICU Hours | 1.001 | 1.001-1.001 | <0.001 |
| Off Pump | 1 | 0.86-1.17 | 0.973 |
| Recent MI | 1.01 | 0.89-1.15 | 0.839 |
| COPD | 1.16 | 0.97-1.38 | 0.094 |
| CVA/TIA | 1.4 | 1.24-1.59 | <0.001 |
| Previous PCI | 1.26 | 1.07-1.48 | 0.006 |
| ACEI/ARB | 1.01 | 0.94-1.09 | 0.777 |
| ASA/anti-platelets | 0.5 | 0.43-0.58 | <0.001 |
| Statins | 0.61 | 0.54-0.7 | <0.001 |
| Beta blockers | 0.7 | 0.62-0.79 | <0.001 |
| RCS1 | 2.14 | 2.09-2.2 | <0.001 |
| RCS2 | 0.94 | 0.91-0.96 | <0.001 |
| RCS3 | 0.88 | 0.87-0.9 | <0.001 |
| RCS4 | 1 | 0.98-1.01 | 0.48 |
| RCS5 | 1 | 0.99-1.01 | 0.908 |
| Constant | 0.19 | 0.11-0.32 | <0.001 |

MACCE, Major cardio-cerebrovascular events; HR, Hazard ratio; CI,Confidence interval; BMI, Body mass index; CKD, Chronic kidney disease; EF, Ejection fraction; SVD,Single vessel disease; VD, Vessel disease; ICU, Intensive care unit; MI, Myocardial infarction; COPD, Chronic obstructive pulmonary disease; CVA, Cerebrovascular accidents; TIA, Tranisent ischemic attack; PCI, Percutaneous coronary intervention; ACIE, Angiotensin converting enzyme inhibitor,; ARB, Angiotensin II receptor blocker; ASA, Aspirin
