# Supplementary figures and images for: The association between different body mass index levels and midterm surgical revascularization outcomes
Source: PLoS One. 2022 Sep 29;17(9):e0274129. doi: 10.1371/journal.pone.0274129 (PMC9522296; doi:10.1371/journal.pone.0274129)

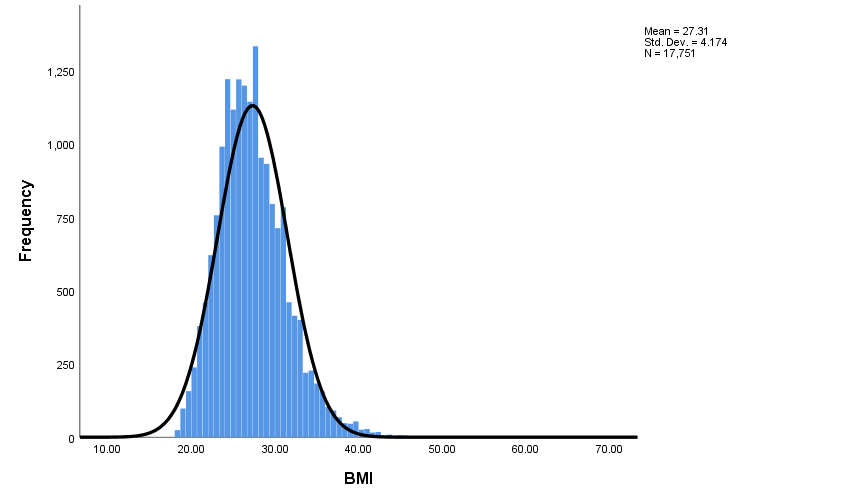

Supplement: S1 Fig — (TIF) [file pone.0274129.s004.tif]
